# Knowledge, attitudes and intention on fertility preservation among breast cancer patients

**DOI:** 10.1038/s41598-023-36377-w

**Published:** 2023-06-14

**Authors:** Jennifer K. Y. Ko, Charleen S. Y. Cheung, Heidi H. Y. Cheng, Sofie S. F. Yung, Ting Ying Ng, Winnie W. Y. Tin, Ho Yan Yuen, Martin H. C. Lam, Ann S. Y. Chan, Sara W. W. Fung, Vivian C. M. Man, Ava Kwong, Ernest H. Y. Ng

**Affiliations:** 1 Department of Obstetrics and Gynecology, Queen Mary Hospital, The University of Hong Kong, 6/F Professorial Block, 102 Pokfulam Road, Hong Kong, Hong Kong; 2 Department of Clinical Oncology, Tuen Mun Hospital, Hong Kong, Hong Kong; 3 Department of Surgery, Pok Oi Hospital, Hong Kong, Hong Kong; 4 Department of Clinical Oncology, Queen Elizabeth Hospital, Hong Kong, Hong Kong; 5 Department of Clinical Oncology, Queen Mary Hospital, Hong Kong, Hong Kong; 6 Department of Surgery, Kwong Wah Hospital, Hong Kong, Hong Kong; 7 Department of Surgery, Queen Mary Hospital, The University of Hong Kong, Hong Kong, Hong Kong

**Keywords:** Oncology, Quality of life, Breast cancer

## Abstract

Breast cancer is the most common cancer in reproductive age women. The aim of this study is to assess the knowledge, attitude and intention on fertility preservation among women diagnosed to have breast cancer. This is a multi-centre cross-sectional questionnaire study. Reproductive age women diagnosed with breast cancer attending Oncology, Breast Surgery and Gynaecology Clinics and support groups were invited to participate. Women filled in paper or electronic form of the questionnaire. 461 women were recruited and 421 women returned the questionnaire. Overall, 181/410 (44.1%) women had heard of fertility preservation. Younger age and higher education level were significantly associated with increased awareness of fertility preservation**.** Awareness and acceptance of the different fertility preservation methods in reproductive age women with breast cancer was suboptimal. However, 46.1% women felt that their fertility concerns affected their decision for cancer treatment in some way.

## Introduction

Breast cancer is the most common cancer in reproductive age women in Hong Kong. Advances in cancer treatment has resulted in high cure rate of breast cancer and an overall five-year relative survival rate of patients with breast cancer of more than 80% in many developed countries^1,2^. Nevertheless, cancer treatment can have adverse effects on the ovaries^3^. To young women, one of the most devastating effects of post cancer treatment is the permanent loss in fertility from premature ovarian insufficiency. With the global trend towards delay in childbearing^4^, many women have not completed their family at cancer diagnosis^5^. Studies have shown that many young cancer patients have a strong desire to have children^6^, and failure to fulfil this desire has been associated with worse mental health^7^.

Guidelines on fertility preservation emphasize the importance of counselling patients on the impact of cancer treatment on their reproductive function and considering fertility preservation for those likely to be affected^8,9^. A questionnaire study on physicians attending two international breast cancer conferences showed that a significant proportion had not consulted available international guidelines on fertility preservation and did not know the different fertility preservation options available in their country^10^. Similarly, local studies conducted in Hong Kong among health care professionals several years ago showed that only 45.6% of them were familiar with fertility preservation. The reasons for clinicians not to refer their patients for fertility preservation included a lack of available time before cancer treatment, considerable risk of cancer recurrence, poor prognosis, financial constraints, need for cancer treatment as top priority at the time, and lack of awareness of such service^11^.

Studies evaluating breast cancer patients’ knowledge, perceptions and needs are generally of small sample size or descriptive in nature. The aim of this study is to assess the knowledge, attitude and intention on fertility preservation among women diagnosed to have breast cancer, so as to better understand their actual overall needs, identify the inadequacies and thereby target improvement on this aspect of oncological care.

## Methods

### Participants

This is a multi-centre cross-sectional questionnaire study on reproductive aged women (18–45 years old at the time of recruitment) who had been diagnosed with breast cancer. Women who could not read Chinese or English were excluded from the study.

Women were recruited from Clinical Oncology Clinics at Queen Mary Hospital, Queen Elizabeth Hospital and Tuen Mun Hospital, Breast Surgery Clinics at Queen Mary Hospital, Kwong Wah Hospital and Pok Oi Hospital, Gynaecology Clinic at Queen Mary Hospital and patient support groups of the Hong Kong Cancer Fund. The questionnaire was also available online on our departmental social media website, so patients seen in other clinics or in the private sector who self-identified themselves as eligible according to the inclusion and exclusion criteria could also have access to the questionnaire and participate in the study. Recruitment period was from September 2020 to February 2022.

Ethics approval was obtained from Institutional Review Board of the University of Hong Kong/ Hospital Authority Hong Kong West Cluster, Kowloon Central Cluster Research Ethics Committee and the New Territories West Cluster Research Ethics Committee. All research was performed in accordance with relevant guidelines and regulations. Informed consent was obtained from all participants. The questionnaire was anonymous with the option for participants to leave their names and phone number if they wished to be contacted by the research team.

### Questionnaire development and distribution

The questionnaire consisted of 50 questions in four sections: (1) background demographic information, (2) knowledge on fertility preservation, (3) attitude and intention on fertility preservation and (4) practice on fertility preservation. The questionnaire was drafted taking reference from publication on similar topics on fertility preservation^12–16^. The survey questions were prepared by fertility specialists with input from clinical oncologists and breast surgeons. The questionnaire was available in traditional Chinese and English. The questionnaire was distributed to a pilot group of 20 patients for assessment of its content, clarity and length before finalised. Minor changes in wordings and corrections in typos were made.

Participants could choose to complete the questionnaire either in paper form which was distributed in the clinic by the attending doctor or research nurse or electronically via a QR code printed on posters, pamphlets or on our Departmental social media. The questionnaire required approximately 20 min to complete. Completed paper questionnaires were returned to the research nurse at the end of the consultation, who inputs the data into the computer. The answers in the electronic questionnaire were automatically stored in an Excel file.

### Sample size

The sample size needed for a confidence interval of 5 with 95% confidence level in a large population of 10,000 is 370. Accounting for incomplete return of questionnaires in 20%, 450 women were needed.

### Statistical analysis

Statistical analysis was performed using SPSS (version 26, IBM Corporation, Armonk, NY) and are mainly descriptive. Comparisons between the groups were made using the Chi-square and Mann–Whitney U-test for categorical and continuous variables respectively. As some patients did not answer all questions, the denominator (n) of different categories were included in the table. P value of < 0.05 was considered statistically significant.

## Results

**Figure 1:**
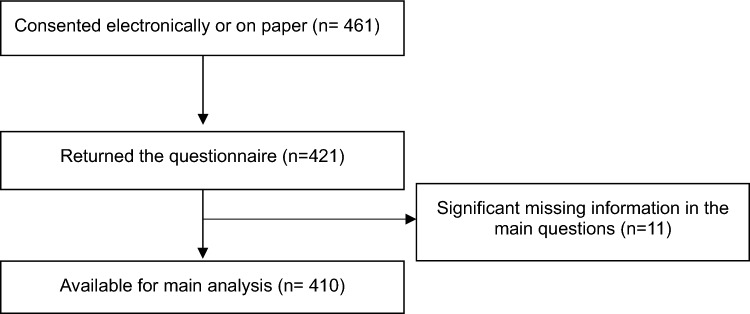
Flow chart of participants.

In total, 461 women consented (on paper or electronically) and 421 questionnaires were returned. Those with significant missing data were excluded in the main analysis. The flow of participants is shown in Fig. 1. As some patients did not answer all questions, the denominator (n) of different categories were included. The background demographics of the participants are shown in Table 1. The mean age of the women is 40.4 ± 4.5 years (mean ± SD).

**Table 1:** Demographic characteristics of participants.

Age (years) (mean $$\pm \mathrm{ SD})$$	40.4 $$\pm$$ 4.5
Ethnicity (n = 421)	
Chinese	400 (95.0)
Non-Chinese Asians	19 (4.5)
Caucasians	2 (0.5)
Education level (n = 421)	
Primary	5 (1.2)
Secondary	194 (46.1)
Associate degree or diploma	35 (8.3)
Tertiary or above	187 (44.4)
Occupation (n = 421)	
Clerical	196 (46.6)
Housewife	105 (24.9)
Professional	66 (15.6)
Manual labour (including hair dresser, domestic helper, saleslady, painter)	30 (7.1)
Unemployed	12 (2.9)
Others	12 (2.9)
Religion (n = 421)	
Buddhism	32 (7.6)
Catholic	22 (5.2)
Christian	82 (19.5)
Nil	283 (67.2)
Others	2 (0.5)
Household income (n = 418)	
Less than HK$10,000	35 (8.4)
HK$10,000–19,999	71 (17.0)
HK$20,000–29,999	98 (23.4)
HK$30,000–39,999	69 (16.5)
More than HK$40,000	145 (34.7)
Marital status (n = 418)	
Married	267 (63.9)
Single, no committed relationship	100 (23.9)
Single, stable partner	51 (12.2)
Sexual orientation (n = 418)	
Heterosexual	395 (94.5)
Homosexual	6 (1.4)
Bisexual	2 (0.5)
Have not decided/ do not want to disclose	15 (3.6)
Pregnant before (n = 417)	217 (52)
Having child(ren) (n = 417)	
None	216 (51.8)
One	103 (24.7)
Two or more	98 (23.5)
Received fertility treatment before (n = 413)	37 (10.4)
Time since diagnosis of breast cancer (months) (n = 413)	
0–6	57 (13.8)
6–12	44 (10.7)
13–24	79 (19.1)
25–60	117 (28.3)
> 60	116 (28.1)
Cancer treatment received/ planned (n = 413)	
Surgery	387 (93.7)
Chemotherapy	283 (68.5)
Radiotherapy	328 (79.4)
Hormonal therapy	145 (35.1)
Targeted therapy	94 (22.8)
Not sure	8 (1.9)
Stage (n = 413)	
Stage I	115 (27.8)
Stage II	164 (39.7)
Stage III	68 (16.5)
Stage IV	9 (2.2)
Not sure	57 (13.8)
Family history of breast or ovarian cancer (n = 413)	69 (16.7)

Data presented as mean $\pm$ standard deviation or number (percentage).

### Knowledge, attitude and intention towards fertility preservation

242/410 (59.0%) women thought that breast cancer treatment would affect fertility, 129/410 (31.5%) women were not sure and 39/410 (9.5%) did not think breast cancer treatment would affect fertility.

**Figure 2:**
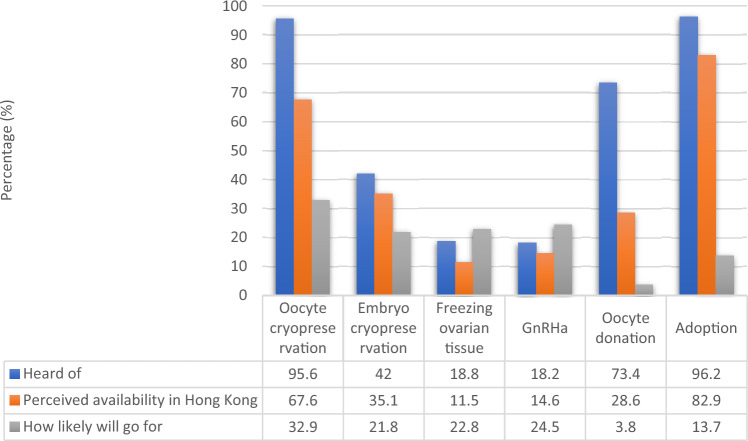
Knowledge and acceptance on the different modes of fertility preservation.

Figure 2 showed the awareness, perceived availability and acceptance on the different modes of fertility preservation.

Overall, 181/410 (44.1%) have heard of fertility preservation. Women who had heard of fertility preservation were significantly younger, had higher education level and had higher monthly family income than women who have not heard of fertility preservation (Table 2). Univariate binary logistic regression showed that age and education level were significantly associated with awareness of fertility preservation (Table 3).

**Table 2:** Patient characteristics and knowledge of fertility preservation.

	Heard of fertility preservation	Not heard of fertility preservation	P value
Age (years)	39.7 ± 4.7	41.0 ± 4.2	0.004*
Ethnicity			0.937
Chinese	172/390 (44.1)	218/390 (55.9)	
Non-Chinese	9/20 (45.0)	11/20 (55.0)	
Education level			0.000*
Tertiary	105/181 (58.0)	76/181 (42.0)	
Below tertiary	76/229 (33.2)	153/229 (66.8)	
Family income			0.001*
≥ HK$30,000	109/210 (51.9)	101/210 (48.1)	
Below HK$30,000	72/200 (36.0)	128/200 (64.0)	
Parity			0.282
Nulliparity	99/212 (46.7)	113/212 (53.3)	
Multiparity	82/198 (41.4)	116/198 (58.6)	

Data presented as mean $\pm$ standard deviation or number (percentage).

**Table 3:** Univariate binary logistic regression analysis of factors in predicting awareness of fertility preservation.

	B	Exp (B), 95% CI	P value
Age	−0.066	0.936, 0.892–0.982	0.007*
Ethnicity	0.103	1.109, 0.426–2.883	0.832
Education level	0.922	2.515, 1.554–4.072	0.000*
Family income	0.215	1.240, 0.777–1.981	0.367
Parity	−0.165	0.848, 0.549–1.309	0.848

*Statistically significant.

**Figure 3:**
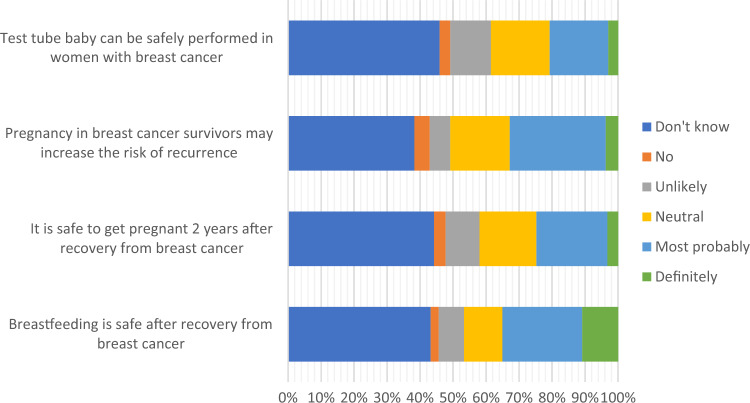
View on in vitro fertilization, pregnancy and breastfeeding. Data presented in bar charts as number (percentage).

121/405 (29.9%) were optimistic that fertility preservation options could lead to a live birth (more than 50% success rates) in cancer survivors. 150/405 (37.0%) thought that it was possible (less than 50% success rates) and 134/405 (33.1%) thought that the overall success was low or that they were still experimental. Figure 3 showed their views on in vitro fertilization, pregnancy and breastfeeding. 37/405 (9.1%) were aware that one needed to be married to use frozen oocytes for assisted reproductive treatment in Hong Kong based on the Code of Practice of the Council on Human Reproductive Technology.

**Figure 4:**
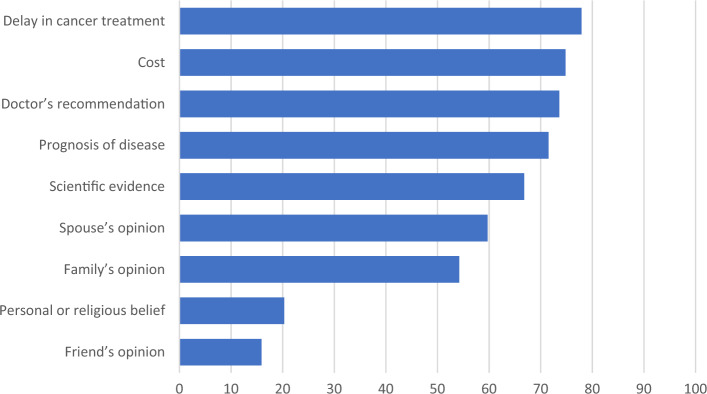
Factors affecting women’s consideration of fertility preservation, n = 389.

In our cohort, having children was important in 92/403 (22.8%) women. The majority was neutral (168/403, 41.7%), and it was not so important in 143/403 (35.5%) women. 54/403 (13.4%) women would like to have (further) children, 244/403 (60.5%) did not want to have (further) children and 105/403 (26.1%) were not sure. Factors affecting their decision of whether to have or not have further children, reasons for wanting or not wanting more information about fertility preservation before starting treatment, and reasons for considering or not considering fertility preservation procedures are shown in Table 4. The factors affecting women’s consideration of fertility preservation is shown in Fig. 4. 66% would not consider fertility preservation before cancer treatment.

**Table 4:** Attitude towards fertility preservation.

1. Factors affecting whether they would like to have or not have further children	n = 403
Personal choice	174 (43.2)
Financial concerns	157 (39.0)
Caring for them if cancer recurs	153 (38.0)
Worry that pregnancy would increase the risk of recurrence	144 (35.7)
Worry that children may have an increased risk of developing cancer	106 (26.3)
Medical concerns (age, already had hysterectomy)	4 (1.0)
2. Reasons for wanting to understand more about fertility preservation before staring cancer treatment	n = 230
This is a human right to reproductive choices	169 (73.5)
I feel more in control	135 (58.7)
I do not want to regret in future	95 (41.3)
I wish to have children in future	52 22.6)
3. Reasons for not wanting more information about fertility preservation before starting cancer treatment	n = 172
I do not have strong wish to have children in future / have kids already	135 (78.5)
I feel overwhelmed by the cancer diagnosis already and this is too complicated for me to understand	27 (15.7)
I do not want to delay treatment as I am not going to do anything	20 (11.6)
These can be discussed later when I recover	25 (14.5)
I believe there will be medical advancement in future making childbearing possible for me	10 5.8)
4. Reasons for considering fertility preservation procedures before starting cancer treatment	n = 135
I may regret in future if I do not take the chance now	79 (58.5)
It is a scientifically feasible option	59 (43.7)
It is a hope for my future	42 (31.1)
Conserving fertility is very important to me	34 (25.2)
Others	2 (1.5)
5. Reasons for not considering fertility preservation procedures before starting cancer treatment	N = 264
I do not want to delay cancer treatment	150 (56.8)
No plans to have children/ completed family	53 (20.1)
It is invasive, I do not want to have additional procedures and related risks	50 (18.9)
I may go through cancer treatment without fertility problems, I may wish to wait and see	22 (8.3)
This is against my cultural, religious belief or personal wish	11 (4.2)
I am doubtful about the success rate and safety of procedures	12 (4.5)
Fertility preservation is too costly	5 (1.9)

Data presented as number (percentage).

When we only included nulliparous women, 113/212 (53.3%) have not heard of fertility preservation. 133/208 (63.9%) would want further information about fertility preservation before their cancer treatment and 80/205 (39.0%) stated that they would consider fertility preservation before cancer treatment.

Overall, 184/386 (47.7%) women did not accept any delay in cancer treatment to attempt fertility preservation. 49/386 (12.7%) and 62/386 (16.1%) accepted 1 week and 2 weeks delay respectively.

127/386 (32.9%) women felt that they should pay for fertility preservation, 167/386 (43.3%) were neutral and 92/386 (23.8%) felt that they should not have to pay for fertility preservation. 326/386 (84.5%) women felt that the consultation should be provided by the government or cost below HK$1000. For the fertility preservation procedure, 177/386 (45.9%) women thought that it should be funded by the government. 92/386 (23.8%) and 88/386 (22.8%) were willing to pay less than HK$10,000 and between HK$10,000–39,999 respectively. Only 4/386 (1.0%) were willing to pay more than HK$70,000.

### Practice of fertility preservation

166/382 (43.5%) women recalled that the doctor had discussed fertility issues with them since cancer diagnosis. If discussion on fertility preservation occurred, the discussion was done by the breast surgeon (116/166, 69.9%) clinical oncologist (96/166, 57.8%), reproductive medicine specialist (18/166, 10.8%) and family doctor (7/166, 4.2%). The discussion on fertility was initiated by the doctor in 111/166 (66.9%) and by the patient 45/166 (27.1%) or her family (10/166, 6.0%) and took place before cancer treatment in 143/166 (86.1%) of women but only after recovery in 22/166 (13.2%) women.

The discussion most commonly included the effect of cancer therapy on fertility (142/166, 85.5%), options of fertility preservation suitable in their situation (55/166, 33.1%), procedure and risks of fertility preservation (33/166, 19.9%), success of fertility preservation (21/166, 12.7%) and cost of fertility preservation (17/166, 10.2%).

176/382 (46.1%) women felt that fertility concerns affected their decision for cancer treatment in some way.

## Discussion

Our study showed that less than half of reproductive age women with breast cancer have heard of fertility preservation. Women who were younger and had higher education level had better awareness on fertility preservation compared to their older and less educated counterparts. Established techniques like oocyte cryopreservation and embryo cryopreservation were more well-known but much fewer women were aware of other fertility preservation methods including ovarian tissue cryopreservation or gonadotrophin releasing hormone agonists during the course of chemotherapy. Acceptance of different fertility preservation methods was generally low.

Fertility preservation allows young women diagnosed with cancer a way to take action when confronted with the potential loss of fertility as a result of cancer and its treatment^17^. On the other hand, having cancer diagnosed is already overwhelming to a young woman. Deciding within a short frame of time to go for fertility preservation and then undergoing the fertility preservation procedures can add further stress at this challenging time^17^. To make informed decisions, women need to understand the effects of cancer treatment on fertility and the available options, although not all women may opt to proceed with fertility preservation. Poor knowledge in fertility preservation has been found to be associated with decisional conflict^18^. In our study, 42.8% women expressed that they did not want further information about fertility preservation before cancer treatment, most commonly because they did not have strong wish to have children in the future. Women in our study were generally older with a mean age of 40 years old. Having children was important in 22.8% of them and only 13.4% women would like to have (further children). In the American Helping Ourselves, Helping Others (HOHO): The Young Women’s Breast Cancer Study, among a prospective cohort of 620 women with breast cancer, 51% were concerned about fertility and in 26% women these concerns affected treatment decisions^19^. In the European HOHO study, among 297 women, 64% were concerned about infertility after treatment and 15% decided not to follow prescribed therapies because of fertility concerns^5^. 66% of women in the American HOHO study already had children at the time they were diagnosed with cancer and 26% reported that they wished to have biologic children in the future^19^. In contrast, there was a higher proportion of women (54%) in the European HOHO study who had not completed their family and among them, a higher proportion (71%) still desired future biologic children following breast cancer^5^. The age and high percentage of women who already had children before cancer (48%) in our study must be taken into account. Nevertheless, 46.1% women in our study felt that their fertility concerns affected their decision for cancer treatment in some way. This ‘contradicting’ view reflects the complexity of the situation. Enhanced collaboration between breast surgeons, clinical oncologists and reproductive medicine specialists is needed so not to jeopardize cancer treatment because of unresolved fertility concerns. It is also important to note that patients’ plans for future children may change, particularly if they are young and childless. In one study, a substantial group of women who did not have a pre-treatment desire for children changed their mind about wanting children after treatment^7^. Better access to information and fertility preservation services with appropriate psychosocial support can empower those who want to further pursue it to go ahead within the narrow window of opportunity. Our practice was still suboptimal with less than half of the women recalling that the doctor had discussed fertility issues with them since cancer diagnosis.

Many factors can affect women’s consideration to whether to pursue fertility preservation. Worry of delaying cancer treatment and the perceived adverse effects of fertility cryopreservation procedures on cancer prognosis can cause reluctance of healthcare professionals to refer the patients for fertility preservation^20,21^. There is common ground here that majority of young patients with breast cancer felt that safety in fertility preservation procedures is paramount and that the procedures should not affect their cancer treatment. Fear of fertility perseveration procedure can deter the patients taking active steps. In the PREFER study, which is an ongoing Italian multicenter, prospective observational study aiming to optimize care and improve knowledge on ovarian function and fertility preservation in young premenopausal breast cancer patients, more than 90% of women were concerned about the potential risk of chemotherapy-induced premature ovarian insufficiency and/or infertility but less than 20% aged ≤ 40 years accepted to undergo cryopreservation, the main reasons for refusal being fear of delaying the initiation of cancer treatment, contraindications to the procedure or lack of interest in future childbearing^22^. Similarly, many of our women did not accept delay in their cancer treatment. With flexible random start ovarian stimulation protocols, around two weeks were still needed for one cycle of ovarian stimulation for oocyte or embryo cryopreservation. This requires prompt and efficient referrals as early as possible within a good oncofertility network. As clinicians, it is important for us to develop safe and effective fertility preservation techniques for patients. A recent meta-analysis showed that controlled ovarian stimulation for oocyte or embryo cryopreservation before starting chemotherapy in young women diagnosed with breast cancer was safe, did not substantially increase the delay in starting chemotherapy and was not associated with detrimental prognostic effect in breast cancer outcomes^23^. Women and healthcare professionals should be reassured that the use of letrozole co-treatment during ovarian stimulation and the random start protocol were equally effective compared with conventional controlled ovarian stimulation, and the overall survival was similar between the women who proceeded to fertility preservation and those who did not^24^. A more recent Canadian study, there were high rates of fertility discussion by surgeons, with fertility preservation being offered to more than 80% women who have not yet completed their families and 47% women who had not completed childbearing underwent fertility preservation. This was likely due to previous knowledge translation intervention and timely referrals^25^.

Fertility preservation is a rapidly expanding field, but is still developing especially in many Asian countries^26,27^. The Asian Society for Fertility Preservation (ASFP) was established in 2015 and the field is becoming more widespread. In Hong Kong, fertility preservation is available in two university affiliated and various private assisted reproduction centres. The pre-requisites of a successful fertility preservation programme include rapid access to the service preferably before the start of gonadotoxic treatment and multidisciplinary team collaboration involving reproductive medicine specialists with expertise on fertility preservation, embryologists and urologists, and strong collaborations with referring specialists including oncologists, haematologists, physicians, surgeons and paediatricians^28^. Local studies conducted in Hong Kong among health care professionals a few years ago have shown that only 45.6% of them were familiar with fertility preservation. There is a need to dedicate more resources to continue to expand the oncofertility network and improve information provision to healthcare professionals so that they can counsel patients appropriately.

Until recently, fertility preservation was only available in Hong Kong as a private service. Financial cost had been identified as a barrier to providing fertility preservation. One cycle of ovarian stimulation with oocyte cryopreservation may cost HK$60,000 to $160,000 in the private sector. Cancer treatment is costly and both physically and emotionally challenging, and financial stress can further be compounded by the loss of work after cancer diagnosis. The newly introduced programme for public-funded fertility preservation allowed patients to undergo the oocyte/ embryo freezing cycle at one-third of the cost of private services. Majority of the women agreed that the fertility preservation procedure should be fully or partially funded by the government.

The provision of reproductive technology procedures, the handling, storing or disposal of gametes or embryos used or intended to be used in connection of a reproductive technology procedure are regulated by the Code of Practice of the Council on Human Reproductive Technology in Hong Kong. Women are required to be legally married to use the frozen oocytes. Only less than 10% women in our study were aware that one needed to be married to use frozen oocytes for assisted reproductive treatment in Hong Kong. The majority either did not know or thought that they do not have to be married to use the cryopreserved oocytes. One should bear in mind the different social aspects and legal regulation in different countries, including whether marriage is a prerequisite for using frozen oocytes, coverage and costs. It would have been interesting to find out the group of women who actually underwent fertility preservation procedures to assess if they have better knowledge and acceptance of the procedure, but whether women actually had fertility preservation was not specifically asked in our questionnaire. Around 10% of women had fertility treatment before, many of which may be related to fertility preservation procedures.

The strength of this paper is that it was a large, multi-centre study involving women of reproductive age with breast cancer from various clinical (oncology, surgical and gynaecological) units as well as in the community. We included women at various stages of breast cancer, including those who have already completed cancer treatment. While this would make the results more representative, this would also include the full spectrum of women who were recently diagnosed and were consulting for fertility preservation and others who may have completed treatment for several years and is on long-term post-treatment follow up at the breast or clinical oncology clinic. However, each woman is unique in their views and circumstances with regard to fertility. We should assess reproductive intentions and tailor reproductive care appropriate for the individual’s intentions.

As it is a self-administered questionnaire, we did not know the true response rate and women who were more concerned with fertility preservation would proceed to complete the questionnaire. Although the questionnaire was available online and patients seen in other clinics or in the private sector who self-identified themselves as eligible according to the inclusion and exclusion criteria could have access to the questionnaire and participate in the study, 410/461 (89%) of the women were recruited and questionnaires were performed by paper (distributed to eligible patients by research staff) or in the presence of doctors or research nurse. In addition to that, some of the participants were approached by research staff at the study sites, private doctors and patient support groups who identified the patients based on the inclusion and exclusion criteria. The percentage of women who self-identified themselves as eligible on social media but in fact may not fit the inclusion and exclusion criteria is likely to be low and unlikely to cause significant bias. As a self-administered questionnaire, women could have misunderstood the actual treatment received or the questions being asked in the questionnaire. The questionnaire was distributed to a pilot group of patients to enhance its content, clarity and length before finalised. Research staff was available at the clinic to clarify any questions the women had when they filled out the questionnaire. The questionnaire also relied on retrospective recall so there may be recall bias but it also reflected the actual information perceived to be important and retained by the patients even years after cancer diagnosis.

## Conclusions

Less than 50% women with breast cancer were aware of fertility preservation and acceptance of the different fertility preservation methods in reproductive age women with breast cancer was low.

## Data Availability

The datasets generated during and/or analysed during the current study are available from the corresponding author on reasonable request.
